# Delivering alcohol screening and alcohol brief interventions within general dental practice: rationale and overview of the evidence

**DOI:** 10.1186/1940-0640-7-S1-A54

**Published:** 2012-10-09

**Authors:** Andrew McAuley, Christine Goodall, Graham Ogden, Simon Shepherd, Karen Cruikshank, Niamh Fitzgerald

**Affiliations:** 1 Public Health Science Division, NHS Health Scotland, Glasgow, Scotland, UK; 2 Department of Dentistry, Glasgow University, Glasgow, Scotland, UK; 3 Department of Dental Surgery, University of Dundee Dental Hospital and School, Dundee, Scotland, UK; 4 Create Consultancy, Ltd., and Robert Gordon University, Aberdeen, Scotland, UK

## 

Alcohol consumption and affordability in the UK has increased over the last 50 years and is associated with a range of adverse oral health outcomes, the most serious of which, oral cancer, is also increasing in incidence. Despite this, routine alcohol screening and brief intervention (SBI) within general dental practice remains uncommon. This review of the literature examined the background and evidence base for undertaking alcohol SBI in general dental practice, including the rationale behind it and the range of issues related to it. Questions addressed include why alcohol consumption is of relevance to dental professionals; what policy context exists for the role of prevention and health promotion in dentistry; what screening tools are appropriate for use within a general dental practice; what evidence of efficacy is there for alcohol SBI in general dental practice; and what barriers and facilitators must be considered before alcohol SBI can be successfully implemented in this setting. We conclude that alcohol SBI in general dental practice, delivered as part of a multidimensional approach to tackling risky alcohol consumption, offer significant potential to improve the oral and general health of the population.

